# Three Cases of Sciatica

**Published:** 1888-01

**Authors:** J. W. Brown

**Affiliations:** Silverton, Colorado


### THREE CASES OF SCIATICA.

By J. W. Brown, M. D., of Silverton, Col.

*Case 1.*—In May, 1884, I was consulted by A. P., about thirty years of age, a tinsmith. He had been suffering over a year from sciatica. His ailment was of a subacute character, but it was subject at times to aggration. As a general thing he was able to follow his occupation, though he was never free from pain, and constantly limped in his walk. He had been subjected to anti-rheumatic remedies, antiphlogistics, and counter irritation by the use of fly blisters, liniments and frictions, and at times, for the allaying of acute suffering, morphine by the hypodermic syringe was used. I had ministered to him several times for this ailment without accomplishing a satisfactory result. He came to me the last time to be relieved from the suffering incident to an aggravation of his trouble. I prescribed one drop of croton oil made into 2 pills, with the extract of gentian as an excipient, and ordered him to take both pills at one dose. The result was a profuse watery discharge from the bowels which brought with it an abundance of scybalous lumps. He was almost entirely relieved of his pain and lameness at once, and remained so for several days, so that I had hopes that a complete cure had been effected. But the pain and lameness gradually returned, and in about ten days he was as bad as before. I again administered the croton oil, with the result of a similar watery discharge and hard lumps of fæcal matter as before, and again there was relief from pain and lameness, but the sciatica again returned after a few days. I concluded that the trouble might be due to the presence of scybala in the rectum or sigmoid colon, the result of constipation, and prescribed a preparation consisting principally of cascara sagrada, which gradually induced a regular daily habit of sufficient discharge from the bowels, and complete cure of the sciatica.

*Case 2.*—W. T., a miner about forty-three years old. He was injured in February, 1883, while handling timber. In tumbling a piece of timber over others, one end of it bobbed up and struck him fairly in the sacroischiatic notch. He fell to the ground in agony and was carried to his bunk in the miners' cabin. I had to make a mountain climb of 1,500 feet to reach him. I used morphine hypodermically and left morphine powders. The next day he was no better. The pain was only slight while he remained perfectly still, and kept the affected limb propped in a certain fixed and semiflexed position, but when he moved the limb, however slightly, a streak of excruciating pain shot down the sciatic nerve and he shrieked with agony. On this second visit I applied a blister a foot long down the thigh over the sciatic nerve, and left instructions for its after-care. I was afraid to use cathartics on account of the patient's inability to manage himself in case catharsis should not bring relief with it, and the use of the croton oil would have been extreme desperation. The blister had no appreciable effect. My patient was no better than the first hour he was injured, and on my third visit I was in a mood to resort to desperate measures. I was getting tired of making a daily mountain climb of 1,500 feet without accomplishing anything, and I went on this third visit armed with two pills containing 1 drop of croton oil. I found my patient propped in the position he had early found to be the easiest. He was not the least improved in his condition, and he was in the constant exertion of a positive mental and muscular effort to keep himself absolutely still. He was becoming worn in consequence of this constant effort to avoid the agony incident to the least motion. I pondered over his case awhile and over the dire catastrophe which would follow the giving of the croton oil if it should not bring relief with the catharsis. I finally resolved that I would administer the croton oil, and that my patient must look after the fæces in such manner as his ingenuity might devise. I took the pills out of my pocket, and saw my patient swallow them, and then I directly retreated down the mountain. The result was wonderful. So soon as it was necessary on account of propriety that my patient should run he was able to do so. He was cured absolutely, immediately, and as if by a miracle. He has never suffered a stitch of pain from that trouble since, and the third day following he went to his regular work. This was a case, probably, of acute inflammation of the sciatic nerve due to a direct blow, and the mode of relief was extreme depletion consequent on the violent catharsis, conjoined with the counter irritation of the croton on the mucous membrane of the bowels.

*Case 3.*—W. A., a miner about forty-two years of age. He had had two attacks of sciatica before, the first lasting four months, the second three months. He consulted me in May for his third attack of sciatica. The pain was not acute, but was a dull, aching variety which was increased in severity by walking. His gait had the characteristic lameness associated with this affection. He said his own bowels were regular. He might have meant that they were regularly constipated, as the fæcal discharge was scybalous and scanty, though he had been having a daily evacuation. I prescribed 1 drop of croton oil in 2 pills, with instructions to take 1 only. He took the pill about 8 o'clock in the evening and was kept busy making excursions to and from the water-closet until the next morning. His pain and lameness disappeared entirely for a time, but gradually returned during the three or four days following, and he took the second pill without being instructed to do so. This pill acted similarly to the first, but not so severely, and he was again relieved for a time only, the pain and lameness gradually returning. I then ordered a pill nightly of nux vomica, hyoscyamus and aloes, which was ineffectual, and I priscribed pills of the extract of cascara sagrada, each 2 grains, with instructions to take enough each night to produce a free daily evacuation. The use of the cascara finally induced a daily habit, and in the course of ten days the sciatica gradually left him, ending in a complete recovery.

These cases are cited to show that sciatica is not always rheumatoid, but is frequently dependent on simple constipation, the correction of which cures the sciatica. The use of the croton oil produces a thorough clearing out of the large intestine and gets rid of all retained lumps of fæcal matter. The return of the sciatica is due to the reaccumulation of scybala, which produces by irritation or pressure on adjacent nerves a reflected neauralgia down the course of the sciatic nerve, sometimes for its whole length.

The use of croton in these cases is not original with the writer. In 1875 Professor Donald McLean, of Ann Arbor, had a case of sciatica of 2 years' standing in the clinic. One drop of croton oil cured his case almost instantaneously, and hence the use of it in the cases above cited. The writer was a student at Ann Arbor during the following session, and Professor McLean related the facts of the case to the class. Regarding the use of the croton as a severe remedy not devoid of possible bad results, the writer was afraid to use it for several years in such cases as above cited, though the opportunity had presented itself several times. Finally necessity compelled its use, and with such remarkable results that the history of these cases has been thought worth a careful study. In the absence of intestinal or stomachic inflammation, subacute or chronic, or hyperæmia conjoined with hyperesthesia, the use of croton is quite safe; but of course, in all cases, the oil should be used only after careful elimination of the possibilities of disaster. —*Journal of the American Medical Association*, Nov. 27, 1887.

